# CAR T-cells in hematologic malignancies: Advances, challenges, and future directions

**DOI:** 10.1016/j.isci.2026.115213

**Published:** 2026-03-04

**Authors:** Karol J. Hernández-Idarraga, Andrea J. Arias-Rozo, Martha L. Arango-Rodríguez, Claudia L. Sossa, Silvia M. Becerra-Bayona

**Affiliations:** 1 Facultad de Ciencias de la Salud, Universidad Autónoma de Bucaramanga - UNAB, Bucaramanga 681003, Colombia; 2 Banco Multitejidos y Centro de Terapias Avanzadas, Clínica FOSCAL Internacional, Floridablanca 681004, Colombia

**Keywords:** oncology, therapeutics

## Abstract

Chimeric antigen receptor (CAR) T-cell therapy has transformed the management of hematologic malignancies, achieving high remission rates in relapsed or refractory B-cell acute lymphoblastic leukemia, large B-cell lymphoma, and multiple myeloma. By redirecting autologous or allogeneic T lymphocytes against tumor-associated antigens such as *CD19* and *TNFRSF17*, CAR T-cells overcome resistance to conventional therapies. Progressive optimization of CAR design—from early constructs to armored and logic-gated platforms—has enhanced persistence, specificity, and safety. Pivotal trials and real-world evidence confirm durable responses, although challenges remain, including cytokine release syndrome, neurotoxicity, manufacturing complexity, high cost, and limited global access. Emerging strategies, such as multi-antigen targeting, gene editing technologies, and *in vivo* CAR delivery, aim to improve efficacy and scalability. Integration of artificial intelligence and point-of-care manufacturing may further streamline production and patient selection. Continued innovation will determine the long-term impact of CAR T-cell therapy as a scalable pillar of precision hematologic oncology.

## Graphical abstract

**Figure:**
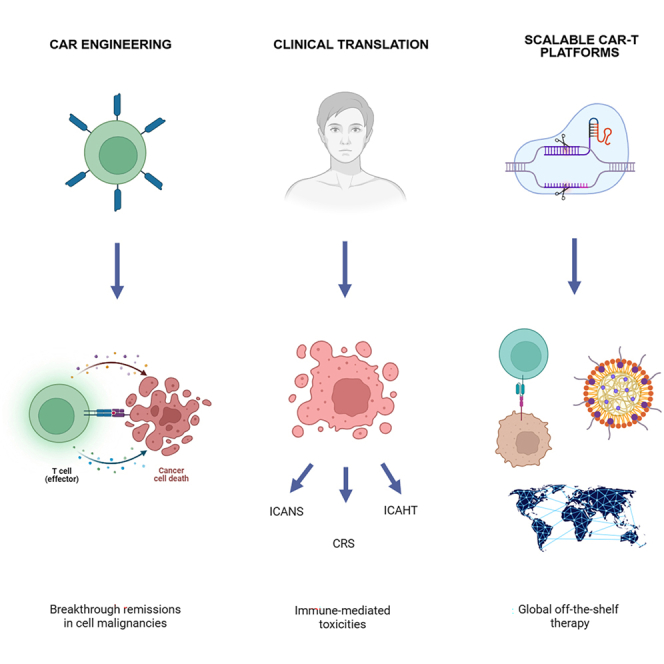

## Introduction

Hematologic malignancies—including acute lymphoblastic leukemia (ALL), large B-cell lymphoma (LBCL), follicular lymphoma (FL), mantle cell lymphoma (MCL), chronic lymphocytic leukemia (CLL), and multiple myeloma (MM)—represent a heterogeneous group of diseases associated with substantial morbidity and mortality despite major advances in chemotherapy, targeted agents, and hematopoietic stem cell transplantation.^1^^,^^2^ While therapeutic outcomes have improved across several B-cell malignancies, a considerable proportion of patients continue to relapse or develop refractory disease, underscoring the need for durable and potentially curative strategies.^3^^,^^4^ Clinical experience and regulatory approvals indicate that chimeric antigen receptor (CAR) T-cell therapy has achieved its greatest maturity and clinical impact in ALL, LBCL, FL, MCL, and MM, where multiple products are approved and incorporated into treatment algorithms.^5^^,^^6^ In contrast, although CAR-T approaches have been explored in CLL, clinical efficacy has been more variable and remains less established than in other B-cell malignancies, justifying a comparatively reduced emphasis in contemporary clinical frameworks.^6^^,^^7^^,^^8^

In relapsed or refractory LBCL, real-world evidence (RWE) consistently demonstrates poor outcomes following failure of standard immunochemotherapy, with limited overall survival (OS) and high rates of early relapse, often inferior to those reported in controlled clinical trials.^9^^,^^10^^,^^11^ These observations highlighted a major unmet clinical need in routine practice and provided a compelling rationale for the development and rapid adoption of CAR T-cell therapy beyond idealized trial populations.^12^^,^^13^ Adoptive cellular immunotherapy has consequently redefined the therapeutic landscape by redirecting T-cell cytotoxicity toward malignant targets through genetic engineering.^14^ CAR T-cell therapy exemplifies this paradigm shift by combining antibody-based antigen recognition with T-cell effector functions, enabling tumor elimination independent of HLA restriction and conventional antigen presentation pathways.^14^

The sequential optimization of CAR architecture—from early constructs containing a single activation domain to later designs incorporating co-stimulatory, cytokine-modulating, or logic-gated elements—has translated into marked improvements in expansion, persistence, and clinical responses in relapsed or refractory B-cell malignancies.^15^ These engineering advances led to multiple regulatory approvals targeting CD19 and B-cell maturation antigen (BCMA; TNFRSF17), producing unprecedented complete response rates and durable remissions in heavily pretreated populations.^4^^,^^16^^,^^17^ Despite these successes, CAR T-cell therapy continues to face critical challenges, including variability in response durability, immune-related toxicities, manufacturing complexity, logistical delays, and high costs that limit equitable global access.^18^ Moreover, relapse mechanisms such as antigen escape and T-cell exhaustion remain major barriers to sustained long-term benefit, emphasizing the need for continued innovation and refinement.^13^^,^^19^

Addressing these limitations requires integrating next-generation CAR designs, optimizing production platforms, and incorporating translational and real-world data to improve patient selection and outcome prediction.^20^^,^^21^^,^^22^ Embedding a real-world perspective from the outset strengthens the clinical relevance of CAR T-cell therapy and aligns ongoing research with the complexities of routine hematologic practice.^23^ This review critically examines the biological principles, structural evolution, and clinical impact of CAR T-cell therapy in hematologic malignancies, integrating translational and real-world perspectives while highlighting emerging strategies and innovations driving the field toward safer, more accessible, and durable cellular immunotherapies.^24^

## Evolution and design of CAR T-cells

### Structural evolution and generational design of CAR T-cells

CAR T-cell therapy represents a paradigm shift in cancer immunotherapy, enabling the redirection of autologous or allogeneic T lymphocytes toward specific tumor-associated antigens through synthetic receptor engineering.^14^ Each CAR construct integrates an extracellular single-chain variable fragment (scFv) that recognizes a tumor antigen, a hinge or spacer domain that provides structural flexibility, a transmembrane domain for membrane anchoring, and one or more intracellular signaling domains that activate T-cell cytotoxicity upon antigen engagement.^18^^,^^25^ The progressive evolution of these elements has given rise to several CAR generations, each designed to enhance activation, persistence, and safety.^26^

**Figure 1:**
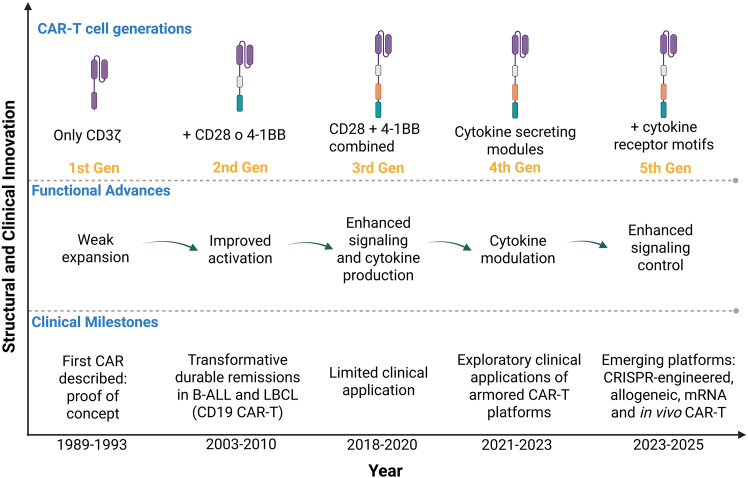
Evolution of CAR T-cell design and key clinical milestones Schematic showing five CAR T-cell therapy generations, from early CD3ζ-only constructs to gene-edited and *in vivo* platforms. Functional and clinical advances are summarized along the timeline. CAR T generations are defined by receptor architecture, rather than clinical efficacy or approval date. Currently approved CD19- and BCMA-directed CAR-T products correspond to second-generation constructs incorporating a single costimulatory domain. Abbreviations: CAR, chimeric antigen receptor; B-ALL: B-cell acute lymphoblastic leukemia; LBCL, large B-cell lymphoma.

The first-generation CARs incorporated a single CD3ζ (CD247) activation motif, which conferred cytotoxic function but limited *in vivo* persistence.^27^ The addition of a co-stimulatory domain such as CD28 or 4-1BB (TNFRSF9) in second-generation CARs markedly improved proliferation and long-term efficacy.^18^^,^^25^^,^^28^^,^^29^^,^^30^^,^^31^ Importantly, second-generation CD19-targeted CAR T-cells marked a transformative clinical milestone, achieving unprecedented complete and durable remissions in both pediatric and adult B-ALL, as well as in diffuse LBCL, even in heavily pretreated and refractory populations. These landmark clinical outcomes redefined therapeutic expectations in relapsed or refractory disease and established second-generation CAR architectures as the backbone of clinically approved CAR T-cell therapies. This profound and reproducible clinical benefit explains why subsequent CAR designs have largely built upon, rather than replaced, second-generation platforms.^32^^,^^33^^,^^34^ Third-generation CARs, combining both CD28 and 4-1BB signals, further enhanced cytokine production and resistance to apoptosis, though clinical benefit over second-generation constructs remains debated.^35^^,^^36^^,^^37^ The introduction of fourth-generation “armored” CARs, equipped with cytokine-secreting or dominant-negative receptor modules—such as IL-12 (*IL12A*/*IL12B*), IL-15 (*IL15*), or PD-1(*PDCD1*)/*CD28* switch receptors—aims to overcome tumor microenvironment–mediated immunosuppression.^17^^,^^26^ More recently, fifth-generation CARs have incorporated cytokine receptor motifs (e.g., IL-2RB-STAT3/5 signaling) or synthetic logic-gated systems designed to improve control and safety^16^^,^^18^ (Figure 1).

The choice of the co-stimulatory domain determines key biological features: CD28-based CARs display rapid expansion and high initial cytotoxicity but shorter persistence, whereas 4-1BB-based CARs expand more slowly yet achieve longer durability and reduced exhaustion.^31^^,^^38^ This balance explains, in part, the differential efficacy and toxicity profiles observed across approved products. For instance, tisagenlecleucel and lisocabtagene maraleucel—both of which are 4-1BB based—show prolonged persistence and lower cytokine release severity compared with CD28-based axicabtagene ciloleucel.^3^^,^^39^

Innovations in CAR design have expanded beyond classical configurations. Dual-target CARs, engineered to recognize two antigens simultaneously (e.g., CD19/CD22 or CD19/CD20; MS4A1), reduce the risk of antigen escape, a major relapse mechanism after single-target therapy.^19^^,^^40^ Armored CARs secrete immune-modulating cytokines (IL-12, IL-18, and IL-15) or incorporate PD-1/CD28 switch receptors, enhancing activity in suppressive microenvironments.^20^ Logic-gated CARs employ AND/OR boolean designs or inhibitory CARs (iCARs) to refine tumor specificity and minimize on-target, off-tumor effects.^41^

In parallel, the manufacturing process has evolved from conventional autologous viral transduction to more flexible and scalable platforms.^42^ Traditional CAR T-cell production uses retroviral or lentiviral vectors for stable gene integration, followed by T-cell activation, expansion, and reinfusion after lymphodepleting conditioning.^17^ However, challenges such as long vein-to-vein time, high cost, and manufacturing failures have stimulated the development of non-viral approaches, including transposon systems (Sleeping Beauty, PiggyBac), CRISPR/Cas9(*CAS9*)-mediated gene insertion, and mRNA electroporation, which allow transient expression and improved safety control.^43^ Moreover, off-the-shelf or allogeneic CAR T-cells, derived from healthy donor T-cells and gene edited to prevent graft-versus-host disease (GvHD), offer the promise of immediate availability and reduced production costs.^26^ Early-phase clinical trials such as UCART19 and ALLO-501A have demonstrated encouraging safety and activity profiles in relapsed/refractory (r/r) B-ALL and LBCL.^3^^,^^44^

Finally, manufacturing optimization and product characterization have become essential for predicting clinical efficacy. Emerging studies employ high-dimensional phenotyping and single-cell analyses to correlate CAR T-cell product composition with outcomes, identifying specific T-cell subsets (e.g., Tscm and Tcm) that associate with durable remission and lower toxicity.^45^^,^^46^These translational insights are expected to guide next-generation CAR development, linking manufacturing state to therapeutic success.^20^

Collectively, these advances in CAR structural design—from first-generation constructs to second-generation platforms incorporating co-stimulatory domains—have defined the foundational engineering principles underlying clinically approved CAR T-cell therapies. However, clinical experience has demonstrated that CAR architecture alone does not fully account for the heterogeneity observed in patient outcomes, suggesting that additional biological determinants beyond receptor design influence therapeutic performance.

### Product profiling and its impact on clinical performance

Beyond CAR structural configuration, growing evidence indicates that the biological composition of the manufactured CAR T-cell product itself plays a critical role in shaping clinical outcomes. High-resolution profiling approaches have revealed substantial heterogeneity at the product level, providing mechanistic insight into inter-patient variability in expansion, persistence, and durability of response. Recent studies have demonstrated that variability in clinical outcomes following CAR T-cell therapy cannot be fully explained by disease burden or patient-related factors alone, highlighting the relevance of intrinsic product-level heterogeneity. High-resolution analyses have shown that CAR T-cell products are composed of multiple cellular subpopulations with distinct transcriptional and functional states and that differences in this composition are associated with variability in expansion kinetics, persistence, and durability of response. In particular, products derived from patients achieving sustained clinical benefit exhibit broader functional diversity than products associated with early relapse or limited expansion.^47^^,^^48^

The differentiation state of T-cells within the infused product has emerged as a critical determinant of CAR T-cell performance. Profiling studies consistently report that products enriched in less differentiated, memory-like T-cell subsets demonstrate superior *in vivo* expansion and long-term persistence, translating into more durable remissions. Conversely, products dominated by more differentiated or terminal effector phenotypes tend to exhibit reduced proliferative capacity and inferior clinical outcomes.^48^^,^^49^ Beyond differentiation, pre-existing exhaustion and chronic activation programs within CAR T-cell products have been linked to impaired therapeutic efficacy. Transcriptomic profiling has identified exhaustion-associated signatures in products from patients with suboptimal responses, suggesting that dysfunctional cellular states may already be imprinted during manufacturing and prior to infusion. These findings underscore that CAR T-cell dysfunction can be, at least in part, product intrinsic, rather than exclusively driven by post-infusion tumor-mediated mechanisms.^50^^,^^51^

Integrated transcriptomic and metabolic profiling further indicates that cellular metabolic fitness influences CAR T-cell persistence and functionality. Products associated with durable responses display metabolic programs consistent with enhanced flexibility and reduced cellular stress, whereas metabolically dysregulated profiles correlate with limited persistence and diminished clinical benefit. These observations position metabolic state as an additional layer through which product composition shapes therapeutic outcomes.^47^^,^^52^^,^^53^ Advances in single-cell RNA sequencing, high-dimensional flow cytometry, and multimodal analytical platforms have enabled increasingly precise mapping of CAR T-cell product compositions. These tools allow direct correlation of cellular phenotypes and transcriptional programs with downstream clinical features, including early expansion dynamics, toxicity profiles, and response durability, providing a more mechanistic understanding of inter-patient variability.^42^^,^^48^^,^^54^

Collectively, these findings support a shift in perspective in which deep product profiling evolves from a retrospective explanatory tool to a potential prospective quality attribute. By informing manufacturing optimization, patient stratification, and rational CAR design, systematic characterization of CAR T-cell products may play an increasingly important role in improving clinical outcomes and advancing personalized cellular immunotherapy.^47^^,^^52^

## Mechanisms of resistance and relapse after CAR T-cell therapy

Although CAR T-cell therapy has achieved remarkable efficacy in r/r B-cell malignancies, up to 50% of patients ultimately relapse, reflecting both tumor-intrinsic and T-cell intrinsic mechanisms of resistance.^18^^,^^40^ Understanding these processes is essential for optimizing next-generation CAR T-cell designs and improving long-term disease control.

### Antigen escape and lineage plasticity

The most frequent mechanism of relapse is antigen loss or modulation of the target molecule. In CD19-directed CAR T-cell therapy, leukemic clones can downregulate or splice the CD19 epitope, produce truncated isoforms lacking the scFv binding site, or undergo lineage switching from B-cell- to myeloid-like phenotypes.^55^^,^^56^ Similarly, in MM, resistance to BCMA-directed CAR T-cells may arise from antigen shedding, mediated by γ-secretase activity or biallelic loss of BCMA.^19^^,^^57^ These findings highlight the need for dual- or multi-antigen targeting strategies such as CD19/CD22, CD19/CD20, or BCMA/GPRC5D CARs, which are currently demonstrating lower relapse rates in early-phase trials.^16^^,^^40^

### T-cell exhaustion and metabolic dysfunction

CAR T-cell persistence strongly correlates with durable remission. However, repeated antigen stimulation, tonic signaling from constitutively active CARs, and exposure to immunosuppressive cytokines (e.g., IL-10; *IL10* and TGF-β) induce T-cell exhaustion characterized by loss of proliferative capacity and expression of inhibitory receptors such as PD-1, TIM-3, and LAG-3.^58^ Transcriptional reprogramming driven by TOX and NR4A family members further enforces this dysfunctional state.^18^^,^^59^ Metabolic stress also contributes to exhaustion: limited glucose and amino acid availability in the tumor niche impairs glycolysis and mitochondrial fitness.^60^ Preclinical models suggest that modulating CAR signaling strength, using cytokine support (IL-15 and IL-21), or engineering CAR-T cells with metabolic reprogramming capacity can delay or reverse exhaustion^46^

### Tumor microenvironment and immune suppression

Although hematologic malignancies lack the dense stromal barriers of solid tumors, they develop immunosuppressive microenvironments rich in regulatory T-cells (Tregs), myeloid-derived suppressor cells (MDSCs), and M2-like macrophages that inhibit CAR T-cell activation^45^ High levels of inhibitory ligands such as PD-L1, IDO, and galectin-9 further attenuate CAR T-cell cytotoxicity.^61^ “Armored” CAR T-cells engineered to secrete IL-12 or express PD-1/CD28 switch receptors can locally reprogram these suppressive niches^20^^,^^62^ Combination approaches with immune checkpoint inhibitors or BTK inhibitors have also shown synergistic activity, improving expansion and persistence in refractory lymphoma and CLL.^3^^,^^19^

### Strategies to overcome resistance

The integration of multi-antigen recognition, gene editing, and adjunctive immunomodulation constitutes the current frontier in overcoming CAR T-cell resistance. Dual-target CAR T-cells mitigate single-antigen escape, whereas allogeneic or stem cell-derived CAR T-cell platforms enable repeated dosing without autologous constraints.^26^^,^^44^ The incorporation of dominant-negative TGF-β receptors, cytokine-releasing modules, or transcriptional rewiring tools (e.g., NR4A deletion) has demonstrated improved functionality in preclinical settings.^63^ Future strategies will likely combine CAR T-cell therapy with checkpoint blockade, small-molecule signaling modulators, or metabolic enhancers, aiming to transform transient responses into long-term immune surveillance.^20^

These resistance mechanisms not only determine the duration of remission but also influence real-world outcomes and patient selection criteria in clinical practice. The following sections, therefore, review the main clinical trials of CAR T-cell therapy in hematologic malignancies, highlighting efficacy benchmarks, toxicity profiles, and ongoing strategies to improve response durability.

## Clinical trials of CAR T-cell therapy in hematologic malignancies

CAR T-cell therapy has transformed the therapeutic landscape of hematologic malignancies, providing effective options for patients with r/r disease who previously had limited treatment alternatives.^4^^,^^17^ In this section, major clinical trials evaluating CD19^−^ and BCMA-targeted CAR T-cell products are summarized, highlighting differences in efficacy, safety, and durability across B-cell malignancies (Table 1).

**Table 1:** Pivotal clinical trials of CAR T-cell therapy in hematologic malignancies

Product	Target	Disease	Phase (trial ID)	Population	ORR (%)	CR (%)	DOR (mo)	PFS/EFS (mo)	Median follow-up (mo)	Reference
Tisagenlecleucel	CD19	B-ALL (pediatric/young adult)	2 (ELIANA, NCT02435849)	r/r ≥ 2L	81	60	9.2	EFS 50	24	Maude et al.^64^
Brexucabtagene autoleucel	CD19	adult B-ALL	2 (ZUMA-3, NCT02614066)	r/r ≥ 2L	71	56	13.0	PFS 11.6	24	Shah et al.^40^
Brexucabtagene autoleucel	CD19	MCL	2 (ZUMA-2, NCT02601313)	r/r ≥ 2L	93	67	25.9	PFS 12.3	35	Wang et al.^65^
Axicabtagene ciloleucel	CD19	LBCL	2 (ZUMA-1, NCT02348216)	r/r ≥ 2L	83	58	11.1	PFS 5.9	27	Locke et al.^66^
Tisagenlecleucel	CD19	LBCL	2 (JULIET, NCT02445248)	r/r ≥ 2L	52	40	10.3	PFS 3.2	40	Schuster et al.^4^
Lisocabtagene maraleucel	CD19	LBCL	2 (TRANSCEND, NCT02631044)	r/r ≥ 2L	73	53	16.0	PFS 6.8	18	Nastoupil et al.^39^
Idecabtagene vicleucel	BCMA	MM	2 (KarMMa, NCT03361748)	r/r ≥ 4L	73	33	10.9	PFS 8.8	24	Munshi et al.^17^
Ciltacabtagene autoleucel	BCMA	MM	1b/2 (CARTITUDE-1, NCT03548207)	r/r ≥ 4L	98	80	21.8	PFS 34.0	27	Berdeja et al.^67^

Summary of key CD19- and BCMA-directed CAR T-cell trials. ORR, overall response rate; CR, complete remission; DOR, duration of response; PFS/EFS, progression-free survival/event-free survival. “Population” indicates the clinical profile of patients included in each trial, specifying disease status (relapsed/refractory) and the number of prior therapy lines (L) received before CAR T-cell infusion. LBCL includes diffuse large B-cell lymphoma, primary mediastinal B-cell lymphoma, and transformed, as defined in pivotal clinical trials.

### CD19-directed CAR T-cells in ALL

Tisagenlecleucel (Kymriah, Novartis) was the first CAR T-cell therapy approved by the FDA in 2017 for pediatric and young adult patients with r/r B-cell ALL. In the pivotal ELIANA trial, an overall response rate (ORR) of 81% and complete remission (CR) of 60% were achieved, with a 12-month event-free survival (EFS) of 50% and OS of 76%.^64^ Cytokine release syndrome (CRS) occurred in approximately 77% of patients, and most events were of grade 1–2 and manageable with tocilizumab and supportive care. Subsequent real-world studies confirmed comparable outcomes, validating the reproducibility of manufacturing and safety outside trial settings.^68^^,^^69^ These findings established tisagenlecleucel as the benchmark therapy for pediatric and young adult B-ALL.

Newer constructs such as brexucabtagene autoleucel (Tecartus, Kite/Gilead) were later approved for adult B-ALL, showing an ORR of 71% and CR of 56% in the ZUMA-3 trial, with median duration of response (DOR) of 13 months.^70^ Long-term follow-up indicated persistence of CAR T-cells beyond two years in some responders, supporting the role of central memory T-cell subsets in durable remission.^19^

### CD19-directed CAR T-cells in LBCL

CAR T-cell therapy has revolutionized treatment for diffuse LBCL, primary mediastinal B-cell lymphoma, and transformed FL, as defined in pivotal clinical trials collectively categorized under LBCL. Axicabtagene ciloleucel (Yescarta, Kite/Gilead) was evaluated in the ZUMA-1 trial, showing an ORR of 83% and CR of 58% in r/r LBCL after ≥2 prior lines of therapy, with median OS of 25.8 months.^66^ CRS occurred in 94% (13% grade ≥3) and immune effector cell-associated neurotoxicity syndrome (ICANS) in 64% (28% grade ≥3), consistent with the CD28 co-stimulatory profile.

Tisagenlecleucel (JULIET trial) and lisocabtagene maraleucel (TRANSCEND trial), both of which are 4-1BB-based constructs, demonstrated lower CRS/ICANS incidence and longer persistence than CD28-based platforms.^4^^,^^39^ These differences underscore the biological relevance of co-stimulatory domain selection, with CD28 providing rapid expansion but shorter durability, and 4-1BB yielding slower proliferation yet greater longevity.^71^

Beyond aggressive B-cell lymphomas, CAR T-cell therapy has also demonstrated robust and clinically meaningful activity in r/r FL, with high response rates and deep remissions observed in patients with multiple prior lines of therapy, including those with early relapse after immunochemotherapy, supporting its integration into treatment algorithms for high-risk FL.^24^^,^^72^

Real-world data corroborate clinical trial outcomes, with consistent response rates and toxicity patterns across multi-center registries.^73^ However, the need for optimal timing, bridging therapy, and patient selection remains critical, as manufacturing failures and early progression before infusion continue to limit eligibility in up to 10%–15% of cases.^74^

### CD19-directed CAR T-cells in CLL

The efficacy of CAR T-cell therapy in CLL has been historically lower than in ALL and DLBCL due to profound immune dysfunction and the suppressive microenvironment characteristic of CLL. In early studies, ORRs ranged from 25% to 45%, with sustained CRs in only a subset of patients.^75^^,^^76^ As a result, CAR T-cell therapy in CLL remains largely investigational and is not yet established as a standard-of-care option, particularly when contrasted with other B-cell malignancies where regulatory approvals have been achieved.^7^^,^^77^ Combination strategies such as CAR T-cell therapy plus ibrutinib have demonstrated improved expansion, reduced toxicity, and enhanced depth of response, likely due to partial reversal of T-cell exhaustion.^78^ Ongoing trials evaluating allogeneic CD19 CAR T-cell and dual-target constructs (CD19/CD20) aim to enhance efficacy in CLL, potentially overcoming resistance mediated by antigen downregulation.^16^

### BCMA-directed CAR T-cells in MM

MM represents the first plasma cell malignancy successfully treated with CAR T-cells targeting BCMA. Idecabtagene vicleucel (Abecma, Bristol Myers Squibb/Bluebird Bio) demonstrated an ORR of 73% and a CR of 33%, with median progression-free survival (PFS) of 8.8 months in heavily pretreated patients.^17^ The Ciltacabtagene autoleucel (Carvykti, Janssen/Legend) trial reported superior results, with an ORR of 98%, a CR of 80%, and 27-month median PFS of 34 months.^67^ Despite remarkable efficacy, late-onset toxicities, prolonged cytopenias, and infections remain frequent, requiring extended monitoring. Registry-based and real-world analyses confirm the reproducibility of these outcomes outside clinical trials while highlighting disease-specific toxicity profiles and durability differences between products, reinforcing the clinical maturity of BCMA-directed CAR T-cell therapy in MM.^9^^,^^33^ Emerging data on alternative targets such as GPRC5D and FcRH5 (*FCRL5*)^20^ suggest potential for next-generation CAR T-cell expansion beyond BCMA, possibly reducing relapse driven by antigen escape.

### Comparative overview and real-world outcomes

Across hematologic malignancies, the ORRs for CD19-directed CAR T-cell therapy range from 60% to 90% and from 70% to 98% for BCMA-targeted constructs. However, the strength of evidence and clinical integration vary substantially by disease, with ALL, LBCL, FL, MCL, and MM representing indications with the most mature clinical data and regulatory validation, whereas CLL remains an area of active investigation.^5^^,^^6^ Durability of remission varies by the co-stimulatory design and disease context, with 4-1BB-based products showing longer persistence but delayed expansion compared with CD28-based constructs. RWE analyses consistently align with pivotal trials, confirming reproducibility in efficacy and toxicity, yet highlight practical barriers including manufacturing delays, variable access, and high cost.^3^^,^^73^ Incorporating patient-centered selection criteria and predictive biomarkers—such as CAR T-cell phenotype, cytokine kinetics, and tumor burden—will be essential to optimize outcomes and resource allocation.

## CAR T-cell therapy toxicities and their management

CAR T-cell therapy has a distinctive safety profile characterized by immune-mediated adverse events resulting from robust T-cell activation and cytokine release. The two most relevant toxicities are CRS and ICANS, which can occur individually or concurrently.^68^^,^^79^ The frequency and severity of these events vary among CAR products, reflecting differences in antigen target, disease burden, and co-stimulatory domain signaling^80^ (Table 2).

**Table 2:** Toxicities associated with CAR T-cell therapy and their management

Toxicity	Onset (days post-infusion)	Pathophysiology/key cytokines	Grading (ASTCT 2019)	Main management	Supportive care
Cytokine release syndrome (CRS)	1–5	IL-6, IFN-γ, TNF-α → endothelial activation, vascular leak	Fever, hypoxia, and vasopressor use	tocilizumab ± corticosteroids	oxygen, fluids, and antipyretics
Immune effector cell-associated neurotoxicity (ICANS)	5–10	BBB disruption, cytokine-mediated neuroinflammation	ICE score, seizures, motor changes, and edema	corticosteroids (dexamethasone); ICU, if severe	seizure prophylaxis, EEG, and neuro-monitoring
Prolonged cytopenias	10–30	myelosuppression, cytokine inhibition of hematopoiesis	–	G-CSF after CRS resolution	transfusion and antimicrobial prophylaxis
Infections	variable	B-cell aplasia, lymphopenia	–	antimicrobial therapy ± IVIG	vaccination and prophylaxis
Other (MAS/HLH-like)	3–10	hyperactivation of macrophages, IFN-γ storm	Ferritin >10,000 ng/mL and organ failure	corticosteroids ± anakinra	ICU management

Summary of major CAR T-cell related toxicities and recommended management according to the ASTCT 2019 consensus criteria.^68^

MAS, macrophage activation syndrome; ICE, immune effector cell-associated encephalopathy, HLH, hemophagocytic lymphohistiocytosis; BBB, blood-brain barrier; CRS, cytokine release syndrome; G-CSF, granulocyte colony-stimulating factor; EEG, electroencephalogram.

### CRS

CRS results from a massive and transient release of proinflammatory cytokines—including IL-6 (*IL6*), IFN-γ (*IFNG*), IL-10, and TNF-α (*TNF*)—triggered by CAR T-cell activation and interaction with target cells.^81^ Clinically, it manifests as fever, hypotension, tachycardia, and hypoxia, typically emerging within the first week after infusion. The ASTCT consensus grading system (2019) classifies CRS severity based on fever, oxygen requirement, and vasopressor use.^82^ Management includes tocilizumab (anti–IL-6 receptor antibody) as first-line therapy, often combined with corticosteroids for persistent or severe cases. Supportive measures—oxygen supplementation, fluid resuscitation, and close monitoring—remain essential. Early recognition and prompt treatment have substantially reduced the incidence of grade ≥3 CRS, which now occurs in <10% of patients as reported in most pivotal trials.^4^^,^^17^^,^^79^

### Immune ICANS

ICANS represents a spectrum of neurocognitive dysfunction associated with endothelial activation and disruption of the blood-brain barrier secondary to cytokine rises.^68^ Symptoms range from headache, aphasia, tremor, and disorientation to seizures or cerebral edema in severe cases. The onset is usually delayed relative to CRS, occurring within days 5–10 post-infusion. ASTCT criteria evaluate mental status changes (ICE score), seizures, motor findings, and cerebral edema to determine grade. Mild ICANS often resolves spontaneously, whereas severe cases require corticosteroids (e.g., dexamethasone) and intensive neurological monitoring. Tocilizumab is generally ineffective for isolated neurotoxicity, underscoring the importance of differentiating CRS- from ICANS-driven pathology.^83^ Incidence varies by product: CD28-based CARs (e.g., axi-cel) show higher rates (up to 60%) than 4-1BB-based constructs (≤30%) due to rapid cytokine kinetics.^39^^,^^79^

### Other toxicities and late complications

In addition to CRS and ICANS, immune effector cell-associated hematotoxicity (ICAHT), infections, cardiovascular complications, and less common inflammatory syndromes are increasingly being recognized as clinically relevant toxicities following CAR T-cell therapy, particularly in the post-acute phase.^84^^,^^85^^,^^86^^,^^87^^,^^88^ Among these, ICAHT has emerged as a major determinant of late morbidity and healthcare utilization. ICAHT is characterized by prolonged or biphasic cytopenias persisting beyond day 30 after CAR T-cell infusion and affects approximately 20%–40% of patients, depending on disease type and CAR construct. Proposed mechanisms include cytokine-mediated hematopoietic stem cell suppression, bone marrow microenvironment injury, and prior treatment-related reserve exhaustion. Clinically, ICAHT is associated with an increased infection risk, transfusion dependence, and delayed immune reconstitution. Management is largely supportive and includes growth factor support after CRS resolution, transfusions, infection prophylaxis, and careful exclusion of marrow relapse or secondary malignancy.^84^^,^^89^

As a direct consequence of prolonged cytopenias and immune dysregulation, infectious complications remain a major contributor to morbidity, with reported incidence ranging from 30% to over 50% within the first six months post-infusion. Risk factors include prolonged neutropenia, B-cell aplasia, hypogammaglobulinemia, corticosteroid exposure, and prior lines of therapy. Evidence-based management relies on antimicrobial prophylaxis, immunoglobulin replacement in selected patients, and vaccination strategies during immune reconstitution phases.^89^^,^^90^

Neurologic complications following CAR T-cell therapy extend beyond classical ICANS. Non-ICANS neurotoxicities—including peripheral neuropathy, cranial nerve involvement, movement disorders, and delayed neuromuscular symptoms—have been increasingly reported, particularly in patients receiving BCMA-directed CAR T-cell products. These events may occur weeks to months after infusion and are thought to involve off-tumor antigen expression, immune-mediated nerve injury, or delayed inflammatory effects. Management is individualized and includes neurological evaluation, symptomatic treatment, and immunosuppression in selected cases.^89^^,^^90^

At the severe end of the inflammatory toxicity spectrum, CAR T-cell-associated hemophagocytic lymphohistiocytosis (CarHLH), also referred to as macrophage activation syndrome (MAS)-like syndrome, represents a rare but life-threatening hyperinflammatory complication, with an estimated incidence of 1%–5%. It is characterized by extreme hyperferritinemia, cytopenias, organ dysfunction, and uncontrolled macrophage activation, often overlapping clinically with severe CRS. Current management strategies include high-dose corticosteroids, IL-1 blockade with anakinra, and aggressive supportive care in intensive settings.^84^^,^^89^

### Preventive strategies and clinical management

Optimization of pre-infusion conditioning, early intervention protocols, and real-time cytokine monitoring have markedly improved safety. Prophylactic tocilizumab or corticosteroids in high-risk patients reduce grade ≥3 CRS without impairing efficacy.^3^^,^^91^ Emerging strategies include next-generation CAR designs with attenuated activation domains, suicide switches, or IL-1 blockade (anakinra) to prevent severe inflammation.^18^ Close multidisciplinary collaboration among hematologists, intensivists, and neurologists is fundamental to mitigating morbidity and improving recovery.

### Clinical outcomes and management evolution

The implementation of standardized grading and management guidelines has reduced fatal toxicity rates to <2% across pivotal studies. Real-world analyses confirm similar safety when managed by experienced multidisciplinary teams.^73^^,^^92^ Continued refinement of CAR design, early biomarker detection (e.g., IL-6 and ferritin), and prophylactic interventions will further enhance safety profiles in upcoming generations of CAR T-cell therapy.

## Clinical applications of CAR T-cell therapy

CAR T-cell therapy, initially developed for r/r B-cell malignancies, is now integrated into clinical practice as a transformative option across selected hematologic cancers. Its application extends beyond the original indications to include investigational and off-label uses in diverse immunophenotypic settings, reflecting both the maturity and adaptability of this platform.^17^^,^^64^^,^^79^

### Approved indications and patient selection

CAR T-cell therapy is currently approved for CD19-directed treatment of r/r B-cell ALL in pediatric and young adults, r/r LBCL in adults, and BCMA-directed therapy for MM after at least four prior lines of treatment.^17^^,^^39^ Eligibility criteria include confirmed CD19 or BCMA antigen expression, adequate organ function, and absence of uncontrolled infection. Prior autologous or allogeneic transplantation does not preclude CAR T-cell therapy, though relapsed timing and performance status strongly influence outcomes. Optimal patient selection remains critical. Individuals with low disease burden and preserved T-cell fitness exhibit improved CAR expansion, lower toxicity, and longer persistence.^1^^,^^45^ Conversely, heavy pretreatment, active infections, or significant comorbidities correlate with manufacturing failure or early relapse. For this reason, multidisciplinary evaluation—including hematology, infectious disease, and critical care—is recommended in specialized centers.

### Emerging and off-label applications

Beyond FDA- and European Medicines Agency (EMA)-approved uses, CAR T-cell therapy is being explored for other hematologic malignancies. In B-cell neoplasms, multi-antigen CAR T-cells targeting CD19/CD22 or CD19/CD20 aim to reduce antigen escape.^19^^,^^40^ In T-cell malignancies, constructs targeting CD5 or CD7 are under development, though the challenge of T-cell fratricide remains substantial.^93^ In acute myeloid leukemia (AML), CAR T-cells directed against CD33, CD123 (*IL3RA*), or CLL-1 (*CLEC12A*) have shown early promise but carry risks of myeloablation due to overlapping expression on normal progenitors.^16^ For Hodgkin lymphoma, CD30 (*TNFRSF8*)-directed CAR T-cell therapy has demonstrated partial and complete responses in small cohorts.^20^ Novel targets such as GPRC5D and FcRH5 in MM represent next-generation approaches potentially addressing BCMA-negative relapse or resistance.^94^

In parallel with these target-expansion strategies, universal (allogeneic) CAR T-cell therapies have emerged as a transformative strategy to overcome limitations associated with patient-specific manufacturing, particularly in heavily pretreated individuals and in T-cell malignancies where autologous products are often unfeasible. Allogeneic CAR T-cells are derived from healthy donors and genetically engineered to disrupt endogenous T-cell receptor (TCR) signaling, thereby reducing the risk of GvHD, while additional modifications aim to mitigate host-versus-graft rejection (HvGR).^95^^,^^96^

Recent clinical advances demonstrate that allogeneic CAR T-cell platforms have progressed beyond proof-of-concept into early-phase clinical efficacy. Products such as UCART19 and ALLO-501 have shown antitumor activity in r/r B-cell malignancies, while CD7- and CD5-directed allogeneic CAR T-cells have enabled targeting of T-cell ALL and T-cell lymphomas by preventing fratricide through antigen knockout or intracellular sequestration strategies. These developments represent a critical expansion of CAR T-cell applicability to disease settings previously inaccessible to autologous approaches.^95^^,^^97^

Nevertheless, current clinical experience indicates that allogeneic CAR T-cell persistence remains shorter than autologous counterparts, largely due to immune-mediated rejection. Strategies such as beta-2-microglobulin (B2M) disruption, HLA-E expression, alternative cell sources (γδ T-cells or invariant natural killer-iNKT cells), and intensified lymphodepletion are actively being explored to improve durability without compromising safety.^95^^,^^98^

### Integration with other therapies and real-world use

CAR T-cell therapy is increasingly being integrated within multimodal treatments. In CLL, concurrent or sequential use of BTK inhibitors (e.g., ibrutinib) enhances CAR T-cell expansion and persistence, possibly via modulation of the tumor microenvironment.^78^^,^^99^ In LBCL, checkpoint blockade and lenalidomide-based maintenance are being evaluated to prolong remission.^3^

CAR T-cell therapy is also used as a bridge to transplantation, especially in patients achieving partial response or MRD-negativity after infusion.^73^ RWE indicates that, when performed in certified centers, CAR T-cell therapy achieves response rates and toxicity profiles comparable to pivotal trials.^3^^,^^73^ However, logistical and economic barriers persist. Median vein-to-vein time remains 4–6 weeks, and up to 10%–15% of candidates may progress or deteriorate before infusion. Cost, infrastructure, and personnel training continue to limit global accessibility, especially in low- and middle-income regions.

### Pediatric versus adult considerations

Pediatric and young adult patients with B-ALL show superior expansion and persistence of CAR T-cells compared with adult patients, largely due to T-cell fitness and disease biology.^64^^,^^100^ Most pediatric responses are deep and durable, with >50% patients maintaining remission beyond 12 months.^101^ In contrast, adults—particularly those heavily pretreated—exhibit shorter persistence and higher relapse rates, often due to T-cell exhaustion and antigen escape.^19^ Long-term survivorship care is emerging as a new dimension of CAR T-cell application, focusing on immune reconstitution, infection prevention, and psychosocial adaptation following treatment success.

### Clinical perspective

CAR T-cell therapy has transitioned from an experimental rescue intervention to a standard-of-care therapy for several hematologic malignancies. Ongoing clinical experience emphasizes patient optimization before infusion, early toxicity management, and long-term follow-up for late effects. In the coming years, the expansion of point-of-care manufacturing, allogeneic “off-the-shelf” platforms, and integration with digital monitoring tools will further consolidate CAR T-cell therapy as a cornerstone of precision hematologic oncology.^102^

## Future perspectives and current limitations

CAR T-cell therapy represents one of the most significant achievements in the field of cellular immunotherapy, offering the possibility of durable remissions in hematologic malignancies previously considered incurable. As this technology matures, efforts have shifted from proof-of-concept toward improving safety, scalability, and accessibility.^17^^,^^79^ The next generation of CAR T-cells is being designed to address the major barriers identified in clinical practice. Armored CARs, capable of secreting cytokines such as IL-12 or IL-15, aim to resist the immunosuppressive tumor microenvironment, while logic-gated constructs and suicide-switch systems enhance control and minimize off-tumor toxicity.^103^^,^^104^ Fifth-generation CARs integrate cytokine receptor signaling and transcriptional tuning to prolong persistence and delay exhaustion.^105^^,^^106^

In parallel with allogeneic approaches, *in vivo* CAR T-cell generation has emerged as a disruptive frontier aimed at simplifying manufacturing and drastically reducing vein-to-vein time. Unlike conventional *ex vivo* platforms, *in vivo* CAR T-cell-based strategies rely on direct delivery of CAR-encoding genetic material—most commonly mRNA or plasmid DNA—into circulating immune cells, using lipid nanoparticles or viral vectors. Preclinical and early translational studies have demonstrated the feasibility of selectively programming endogenous T-cells within patients, achieving functional CAR expression and antitumor activity without individualized cell manufacturing.^95^^,^^98^

Early *in vivo* CAR T-cell studies highlight several potential advantages, including reduced production costs, faster treatment initiation, and broader global accessibility. However, significant challenges remain, particularly regarding targeting specificity, transduction efficiency, and control over CAR T-cell expansion and persistence. Off-target transfection and uncontrolled immune activation remain key safety concerns, underscoring the need for refined delivery systems and tightly regulated expression cassettes.^95^^,^^97^

Collectively, universal and *in vivo* CAR T-cell platforms represent complementary strategies addressing the logistical, economic, and biological constraints of current CAR T-cell therapy. While both approaches have demonstrated promising early clinical signals, their long-term success will depend on achieving a balance between scalability, safety, and sustained antitumor efficacy. Ongoing trials and technological refinements are expected to clarify their positioning within future treatment algorithms for hematologic malignancies.^95^^,^^98^

Emerging gene-editing technologies such as CRISPR/Cas9, base editing, and epigenetic modulation are enabling the creation of “universal” allogeneic CAR T-cell products, eliminating the need for patient-specific manufacturing.^107^ Moreover, integration of artificial intelligence (AI) and bioinformatics into manufacturing and patient monitoring could accelerate product release, predict toxicity, and refine patient selection using real-time analytics.^108^^,^^109^ These strategies are expected to reduce production variability and costs—a critical step toward global accessibility.

Despite these advances, several limitations persist. The incidence of severe CRS and ICANS, although decreasing, continues to pose significant clinical challenges that require highly specialized multidisciplinary management. Relapse mechanisms, including antigen escape and T-cell exhaustion, continue to restrict long-term efficacy, highlighting the need for improved CAR designs and combination therapies.^110^^,^^111^ Economic and logistical constraints remain major obstacles. Manufacturing is costly and centralized, with median vein-to-vein times exceeding four weeks and production failures occurring in 5%–10% of cases.^112^ The global availability of CAR T-cell therapy is heavily skewed toward high-income countries, with limited access across Latin America, Africa, and parts of Asia. Addressing these inequities will require decentralized point-of-care manufacturing, international cooperation, and public-private partnerships to promote technology transfer and local training.

Secondary primary malignancies (SPMs) after CAR T-cell therapy are increasingly being recognized as a clinically relevant, but generally infrequent, long-term event. A recent systematic review and meta-analysis of 5,517 patients across 18 trials and 7 real-world studies estimated an overall SPM rate of ∼5.8% at a median 21.7 months of follow-up, with hematologic SPMs predominating and T-cell malignancies remaining rare (∼0.09%). Importantly, no increased risk versus standard-of-care was observed in randomized comparisons, suggesting that disease biology and prior therapy exposure may be stronger drivers than the CAR construct itself.^113^ The FDA has investigated a limited number of CAR-associated T-cell malignancies, only a subset of which contained the CAR transgene in tumor cells. Possible mechanisms include insertional mutagenesis, pre-existing clonal hematopoiesis, and cumulative genotoxic exposure from prior cytotoxic therapy. Although causality remains uncertain, these observations underscore the need for systematic molecular surveillance and registry-based pharmacovigilance.^114^ Current evidence supports maintaining long-term follow-up per FDA/EMA guidance (up to 15 years), structured SPM reporting in registries, and targeted molecular analyses (integration-site mapping and single-cell profiling) in secondary events. Future vector designs leveraging non-viral or site-directed integration and integrated safety switches may further mitigate this theoretical risk while preserving therapeutic potency.^113^^,^^114^^,^^115^

Finally, the lack of long-term follow-up data hampers a comprehensive evaluation of late adverse events, immune reconstitution, and secondary malignancies. Establishing multicenter registries and standardized reporting frameworks is essential to harmonize outcomes and compare products fairly across regions. In summary, the future of CAR T-cell therapy depends on its ability to evolve beyond individualized, high-cost interventions toward a more universal, safe, and accessible platform. Ongoing innovations in gene editing, *in vivo* delivery, and AI-driven analytics are expected to redefine the boundaries of cellular therapy, moving closer to sustainable and equitable implementation in hematologic oncology.^116^

## Conclusion

CAR T-cell therapy has redefined the therapeutic paradigm of hematologic malignancies, offering durable responses in patients who had exhausted conventional treatments. The evidence accumulated through pivotal and real-world studies demonstrates that targeting CD19 and BCMA antigens can induce deep and sustained remissions, validating the potential of adoptive cellular immunotherapy as a standard of care in specific clinical settings. Despite this progress, CAR T-cell therapy remains a complex and resource-intensive treatment, with limitations in long-term efficacy, safety, and global accessibility. Relapse due to antigen loss or T-cell exhaustion, together with manufacturing delays and high costs, continues to challenge widespread implementation. Ongoing advances in molecular engineering, gene editing tools, *in vivo* delivery strategies, and AI-supported manufacturing are expected to enhance product consistency, reduce costs, and expand indications to new hematologic malignancies. Ultimately, the success of CAR T-cell therapy will depend not only on its biological innovation but also on its equitable integration into healthcare systems worldwide. As these challenges are progressively addressed, CAR T-cell therapy will continue to evolve from being a highly specialized rescue intervention to a scalable and transformative pillar of personalized cancer treatment.

## Author contributions

K.J.H.-I., A.J.A.-R., M.L.A.-R., C.L.S., and S.M.B.-B. participated in the conception and design of the study; K.J.H.-I. and S.M.B.-B. designed the methodology; K.J.H.-I. and A.J.A.-R. drafted the manuscript; M.L.A.-R., C.L.S., and S.M.B.-B. critically reviewed the manuscript; K.J.H.-I. and A.J.A.-R. wrote the original draft; M.L.A.-R., C.L.S., and S.M.B.-B. revised and edited the final version. All authors approved the version to be submitted.

## Declaration of interests

The authors declare that no conflict of interest could be perceived as prejudicing the impartiality of the review reported.

## Declaration of generative AI and AI-assisted technologies in the writing process

During the preparation of this work, the authors used ChatGPT to improve the readability and language of the manuscript. After using this tool/service, the authors reviewed and edited the content as needed and take full responsibility for the content of the published article.

## References

[1] Dagar G., Gupta A., Masoodi T., Nisar S., Merhi M., Hashem S., Chauhan R., Dagar M., Mirza S., Bagga P. (2023). Harnessing the potential of CAR-T cell therapy: progress, challenges, and future directions in hematological and solid tumor treatments. J. Transl. Med..

[2] Oluwole O.O., Bouabdallah K., Muñoz J., De Guibert S., Vose J.M., Bartlett N.L., Lin Y., Deol A., McSweeney P.A., Goy A.H. (2021). Prophylactic corticosteroid use in patients receiving axicabtagene ciloleucel for large B-cell lymphoma. Br. J. Haematol..

[3] Locke F.L., Miklos D.B., Jacobson C.A., Perales M.A., Kersten M.J., Oluwole O.O., Ghobadi A., Rapoport A.P., McGuirk J., Pagel J.M. (2022). Axicabtagene Ciloleucel as Second-Line Therapy for Large B-Cell Lymphoma. N. Engl. J. Med..

[4] Schuster S.J., Bishop M.R., Tam C.S., Waller E.K., Borchmann P., McGuirk J.P., Jäger U., Jaglowski S., Andreadis C., Westin J.R. (2019). Tisagenlecleucel in Adult Relapsed or Refractory Diffuse Large B-Cell Lymphoma. N. Engl. J. Med..

[5] Minson A., Hamad N., Cheah C.Y., Tam C., Blombery P., Westerman D., Ritchie D., Morgan H., Holzwart N., Lade S. (2024). CAR T cells and time-limited ibrutinib as treatment for relapsed/refractory mantle cell lymphoma: the phase 2 TARMAC study. Blood.

[6] Fox C.P., Townsend W., Gribben J.G., Menne T., Kalakonda N., Williams P., Toron F., Tyas E., Cooper M., Rickards J., Radford J. (2024). Real-world outcomes of patients with relapsed/refractory large B-cell lymphoma receiving second-line therapy in England. EJHaem.

[7] Lim N.A., Sun Y., Tan J.Y., Lim R.M.H., Tan Y.H., Ng L.C.K., Lim F.L.W.I., Goh Y.T., Hoe J.T.M., Chiang J. (2025). Outcomes in relapsed/refractory diffuse large B-cell lymphoma in an Asian tertiary cancer center: real-world interventions as benchmark for novel therapy. ESMO Real World Data and Digital Oncology.

[8] Testa U., Pelosi E., Castelli G., Fresa A., Laurenti L. (2024). CAR-T Cells in Chronic Lymphocytic Leukemia. Mediterr. J. Hematol. Infect. Dis..

[9] Nastoupil L.J., Andersen C.R., Ayers A., Wang Y., Habermann T.M., Chihara D., Kahl B.S., Link B.K., Koff J.L., Cohen J.B. (2025). Real-World Effectiveness of Chemoimmunotherapy and Novel Therapies for Patients With Relapsed/Refractory Aggressive Large B-Cell Lymphoma. Clin. Lymphoma Myeloma Leuk..

[10] Shadman M., Harper J.S., Bokun A., Xu C., Lin P., Graf G., Lu X. (2025). Real-world treatment patterns and clinical outcomes among patients with diffuse large B-cell lymphoma in a US healthcare claims database. Blood Cancer J..

[11] Jiang V., Lee W., Zhang T., Jordan A., Yan F., Cai Q., McIntosh J., Vargas J., Liu Y., Wang M. (2024). The CDK9 inhibitor enitociclib overcomes resistance to BTK inhibition and CAR-T therapy in mantle cell lymphoma. Biomark. Res..

[12] Morschhauser F., Dahiya S., Palomba M.L., Martin Garcia-Sancho A., Reguera Ortega J.L., Kuruvilla J., Jäger U., Cartron G., Izutsu K., Dreyling M. (2024). Lisocabtagene maraleucel in follicular lymphoma: the phase 2 TRANSCEND FL study. Nat. Med..

[13] Thiruvengadam S.K., Ahn K.W., Patel J., Lian Q., Hertzberg M., Epperla N., Metheny L., Hong S., Jain T., Aljurf M. (2025). CD19 Directed CAR T Therapy for Transformed Follicular Lymphoma: A CIBMTR Analysis. Am. J. Hematol..

[14] Adusumilli P.S., Zauderer M.G., Rivière I., Solomon S.B., Rusch V.W., O'Cearbhaill R.E., Zhu A., Cheema W., Chintala N.K., Halton E. (2021). A Phase I Trial of Regional Mesothelin-Targeted CAR T-cell Therapy in Patients with Malignant Pleural Disease, in Combination with the Anti-PD-1 Agent Pembrolizumab. Cancer Discov..

[15] Zheng Z., Li S., Liu M., Chen C., Zhang L., Zhou D. (2023). Fine-Tuning through Generations: Advances in Structure and Production of CAR-T Therapy. Cancers (Basel).

[16] Huang H., Yu L., Weng H., Zhang W., Wang Z., Wang L., Huang H. (2024). Advances in CAR-T cell therapy for hematologic and solid malignancies: latest updates from 2024 ESMO Congress. J. Hematol. Oncol..

[17] Munshi N.C., Anderson L.D., Shah N., Madduri D., Berdeja J., Lonial S., Raje N., Lin Y., Siegel D., Oriol A. (2021). Idecabtagene Vicleucel in Relapsed and Refractory Multiple Myeloma. N. Engl. J. Med..

[18] Rafiq S., Hackett C.S., Brentjens R.J. (2020). Engineering strategies to overcome the current roadblocks in CAR T cell therapy. Nat. Rev. Clin. Oncol..

[19] Tao Z., Chyra Z., Kotulová J., Celichowski P., Mihályová J., Charvátová S., Hájek R. (2024). Impact of T cell characteristics on CAR-T cell therapy in hematological malignancies. Blood Cancer J..

[20] Zhou D., Zhu X., Xiao Y. (2024). CAR-T cell combination therapies in hematologic malignancies. Exp. Hematol. Oncol..

[21] Ahmed G., Alsouqi A., Szabo A., Samples L., Shadman M., Awan F.T., Rojek A.E., Riedell P.A., Iqbal M., Fenske T.S. (2024). CAR T-cell therapy in mantle cell lymphoma with secondary CNS involvement: a multicenter experience. Blood Adv..

[22] Nie E.H., Su Y.J., Baird J.H., Agarwal N., Bharadwaj S., Weng W.K., Smith M., Dahiya S., Han M.H., Dunn J.E. (2024). Clinical features of neurotoxicity after CD19 CAR T-cell therapy in mantle cell lymphoma. Blood Adv..

[23] Argnani L., Broccoli A., Pellegrini C., Fabbri A., Puccini B., Bruna R., Tisi M.C., Masia F., Flenghi L., Nizzoli M.E. (2022). Real-world Outcomes of Relapsed/Refractory Diffuse Large B-cell Lymphoma Treated With Polatuzumab Vedotin-based Therapy. HemaSphere.

[24] Epstein-Peterson Z.D., Lionel A.C., Joseph A., Drill E., Atallah-Yunes S.A., Brooks T.R., Chong E.A., Chong E.R., Dela Cruz J., Frank M.J. (2025). Treatment and outcomes of progression of disease post-CAR T-cell therapy in mantle cell lymphoma: a multicenter analysis. Blood Adv..

[25] Cherkassky L., Morello A., Villena-Vargas J., Feng Y., Dimitrov D.S., Jones D.R., Sadelain M., Adusumilli P.S. (2016). Human CAR T cells with cell-intrinsic PD-1 checkpoint blockade resist tumor-mediated inhibition. J. Clin. Investig..

[26] Lu W., Wei Y., Cao Y., Xiao X., Li Q., Lyu H., Jiang Y., Zhang H., Li X., Jiang Y. (2021). CD19 CAR-T cell treatment conferred sustained remission in B-ALL patients with minimal residual disease. Cancer Immunol. Immunother..

[27] Haynes N.M., Snook M.B., Trapani J.A., Cerruti L., Jane S.M., Smyth M.J., Darcy P.K. (2001). Redirecting mouse CTL against colon carcinoma: superior signaling efficacy of single-chain variable domain chimeras containing TCR-zeta vs Fc epsilon RI-gamma. J. Immunol..

[28] Grupp S.A., Kalos M., Barrett D., Aplenc R., Porter D.L., Rheingold S.R., Teachey D.T., Chew A., Hauck B., Wright J.F. (2013). Chimeric antigen receptor-modified T cells for acute lymphoid leukemia. N. Engl. J. Med..

[29] Zhang M., Chen D., Fu X., Meng H., Nan F., Sun Z., Yu H., Zhang L., Li L., Li X. (2022). Autologous Nanobody-Derived Fratricide-Resistant CD7-CAR T-cell Therapy for Patients with Relapsed and Refractory T-cell Acute Lymphoblastic Leukemia/Lymphoma. Clin. Cancer Res..

[30] Barros L.R.C., Couto S.C.F., da Silva Santurio D., Paixão E.A., Cardoso F., da Silva V.J., Klinger P., Ribeiro P.d.A.C., Rós F.A., Oliveira T.G.M. (2022). Systematic Review of Available CAR-T Cell Trials around the World. Cancers (Basel).

[31] Long A.H., Haso W.M., Shern J.F., Wanhainen K.M., Murgai M., Ingaramo M., Smith J.P., Walker A.J., Kohler M.E., Venkateshwara V.R. (2015). 4-1BB costimulation ameliorates T cell exhaustion induced by tonic signaling of chimeric antigen receptors. Nat. Med..

[32] Laetsch T.W., Maude S.L., Rives S., Hiramatsu H., Bittencourt H., Bader P., Baruchel A., Boyer M., De Moerloose B., Qayed M. (2023). Three-Year Update of Tisagenlecleucel in Pediatric and Young Adult Patients With Relapsed/Refractory Acute Lymphoblastic Leukemia in the ELIANA Trial. J. Clin. Oncol..

[33] Wang M., Munoz J., Goy A., Locke F.L., Jacobson C.A., Hill B.T., Timmerman J.M., Holmes H., Jaglowski S., Flinn I.W. (2023). Three-Year Follow-Up of KTE-X19 in Patients With Relapsed/Refractory Mantle Cell Lymphoma, Including High-Risk Subgroups, in the ZUMA-2 Study. J. Clin. Oncol..

[34] Nastoupil L.J., Bonner A., Wang P., Almuallem L., Desai J., Farazi T., Kumar J., Dahiya S. (2025). Matching-adjusted indirect comparison of efficacy and safety of lisocabtagene maraleucel and mosunetuzumab for the treatment of third-line or later relapsed or refractory follicular lymphoma. Exp. Hematol. Oncol..

[35] Tang X.Y., Sun Y., Zhang A., Hu G.L., Cao W., Wang D.H., Zhang B., Chen H. (2016). Third-generation CD28/4-1BB chimeric antigen receptor T cells for chemotherapy relapsed or refractory acute lymphoblastic leukaemia: a non-randomised, open-label phase I trial protocol. BMJ Open.

[36] Enblad G., Karlsson H., Gammelgård G., Wenthe J., Lövgren T., Amini R.M., Wikstrom K.I., Essand M., Savoldo B., Hallböök H. (2018). A Phase I/IIa Trial Using CD19-Targeted Third-Generation CAR T Cells for Lymphoma and Leukemia. Clin. Cancer Res..

[37] Till B.G., Jensen M.C., Wang J., Qian X., Gopal A.K., Maloney D.G., Lindgren C.G., Lin Y., Pagel J.M., Budde L.E. (2012). CD20-specific adoptive immunotherapy for lymphoma using a chimeric antigen receptor with both CD28 and 4-1BB domains: pilot clinical trial results. Blood.

[38] Guedan S., Posey A.D., Shaw C., Wing A., Da T., Patel P.R., McGettigan S.E., Casado-Medrano V., Kawalekar O.U., Uribe-Herranz M. (2018). Enhancing CAR T cell persistence through ICOS and 4-1BB costimulation. JCI Insight.

[39] Abramson J.S., Palomba M.L., Gordon L.I., Lunning M., Wang M., Arnason J., Purev E., Maloney D.G., Andreadis C., Sehgal A. (2024). Two-year follow-up of lisocabtagene maraleucel in relapsed or refractory large B-cell lymphoma in TRANSCEND NHL 001. Blood.

[40] Shah N.N., Highfill S.L., Shalabi H., Yates B., Jin J., Wolters P.L., Ombrello A., Steinberg S.M., Martin S., Delbrook C. (2020). CD4/CD8 T-Cell Selection Affects Chimeric Antigen Receptor (CAR) T-Cell Potency and Toxicity: Updated Results From a Phase I Anti-CD22 CAR T-Cell Trial. J. Clin. Oncol..

[41] Bangayan N.J., Wang L., Burton Sojo G., Noguchi M., Cheng D., Ta L., Gunn D., Mao Z., Liu S., Yin Q. (2023). Dual-inhibitory domain iCARs improve the efficiency of the AND-NOT gate CAR T strategy. Proc. Natl. Acad. Sci. USA.

[42] Cadinanos-Garai A., Flugel C.L., Cheung A., Jiang E., Vaissié A., Abou-El-Enein M. (2025). High-dimensional temporal mapping of CAR T cells reveals phenotypic and functional remodeling during manufacturing. Mol. Ther..

[43] Abdo L., Batista-Silva L.R., Bonamino M.H. (2025). Cost-effective strategies for CAR-T cell therapy manufacturing. Mol. Ther. Oncol..

[44] Benjamin R., Jain N., Maus M.V., Boissel N., Graham C., Jozwik A., Yallop D., Konopleva M., Frigault M.J., Teshima T. (2022). UCART19, a first-in-class allogeneic anti-CD19 chimeric antigen receptor T-cell therapy for adults with relapsed or refractory B-cell acute lymphoblastic leukaemia (CALM): a phase 1, dose-escalation trial. Lancet Haematol..

[45] Gill S., Vides V., Frey N.V., Hexner E.O., Metzger S., O'Brien M., Hwang W.T., Brogdon J.L., Davis M.M., Fraietta J.A. (2022). Anti-CD19 CAR T cells in combination with ibrutinib for the treatment of chronic lymphocytic leukemia. Blood Adv..

[46] You J., Yang X., Zhao J., Chen H., Tang Y., Ouyang D., Liu Y., Wang Y., Xie S., Chen Y. (2025). Enhancing CAR-T Cell Metabolic Fitness and Memory Phenotype for Improved Efficacy against Hepatocellular Carcinoma. Int. J. Biol. Sci..

[47] Bai Z., Feng B., McClory S.E., de Oliveira B.C., Diorio C., Gregoire C., Tao B., Yang L., Zhao Z., Peng L. (2024). Single-cell CAR T atlas reveals type 2 function in 8-year leukaemia remission. Nature.

[48] Sheih A., Voillet V., Hanafi L.A., DeBerg H.A., Yajima M., Hawkins R., Gersuk V., Riddell S.R., Maloney D.G., Wohlfahrt M.E. (2020). Clonal kinetics and single-cell transcriptional profiling of CAR-T cells in patients undergoing CD19 CAR-T immunotherapy. Nat. Commun..

[49] Deng Q., Han G., Puebla-Osorio N., Ma M.C.J., Strati P., Chasen B., Dai E., Dang M., Jain N., Yang H. (2020). Characteristics of anti-CD19 CAR T cell infusion products associated with efficacy and toxicity in patients with large B cell lymphomas. Nat. Med..

[50] Bai Z., Lundh S., Kim D., Woodhouse S., Barrett D.M., Myers R.M., Grupp S.A., Maus M.V., June C.H., Camara P.G. (2021). Single-cell multiomics dissection of basal and antigen-specific activation states of CD19-targeted CAR T cells. J. Immunother. Cancer.

[51] Chen G.M., Chen C., Das R.K., Gao P., Chen C.H., Bandyopadhyay S., Ding Y.Y., Uzun Y., Yu W., Zhu Q. (2021). Integrative Bulk and Single-Cell Profiling of Premanufacture T-cell Populations Reveals Factors Mediating Long-Term Persistence of CAR T-cell Therapy. Cancer Discov..

[52] Rezvan A., Romain G., Fathi M., Heeke D., Martinez-Paniagua M., An X., Bandey I.N., Montalvo M.J., Adolacion J.R.T., Saeedi A. (2024). Identification of a clinically efficacious CAR T cell subset in diffuse large B cell lymphoma by dynamic multidimensional single-cell profiling. Nat. Cancer.

[53] Peinelt A., Bremm M., Kreyenberg H., Cappel C., Banisharif-Dehkordi J., Erben S., Rettinger E., Jarisch A., Meisel R., Schlegel P.G. (2022). Monitoring of Circulating CAR T Cells: Validation of a Flow Cytometric Assay, Cellular Kinetics, and Phenotype Analysis Following Tisagenlecleucel. Front. Immunol..

[54] Filosto S., Vardhanabhuti S., Canales M.A., Poiré X., Lekakis L.J., de Vos S., Portell C.A., Wang Z., To C., Schupp M. (2024). Product Attributes of CAR T-cell Therapy Differentially Associate with Efficacy and Toxicity in Second-line Large B-cell Lymphoma (ZUMA-7). Blood Cancer Discov..

[55] Lin H., Yang X., Ye S., Huang L., Mu W. (2024). Antigen escape in CAR-T cell therapy: Mechanisms and overcoming strategies. Biomed. Pharmacother..

[56] Ruella M., Barrett D.M., Kenderian S.S., Shestova O., Hofmann T.J., Perazzelli J., Klichinsky M., Aikawa V., Nazimuddin F., Kozlowski M. (2016). Dual CD19 and CD123 targeting prevents antigen-loss relapses after CD19-directed immunotherapies. J. Clin. Investig..

[57] Zhou X., Rasche L., Kortüm K.M., Mersi J., Einsele H. (2023). BCMA loss in the epoch of novel immunotherapy for multiple myeloma: from biology to clinical practice. Haematologica.

[58] Zhu X., Li Q., Zhu X. (2022). Mechanisms of CAR T cell exhaustion and current counteraction strategies. Front. Cell Dev. Biol..

[59] Pei M., Chai W., Wang X., Duan Y., Wang H., Xi Y., Mou W., Wang W., Chen X., Zhang H. (2022). The transcription factor TOX is involved in the regulation of T-cell exhaustion in neuroblastoma. Immunol. Lett..

[60] Zhao S., Peralta R.M., Avina-Ochoa N., Delgoffe G.M., Kaech S.M. (2021). Metabolic regulation of T cells in the tumor microenvironment by nutrient availability and diet. Semin. Immunol..

[61] Sideras K., de Man R.A., Harrington S.M., Polak W.G., Zhou G., Schutz H.M., Pedroza-Gonzalez A., Biermann K., Mancham S., Hansen B.E. (2019). Circulating levels of PD-L1 and Galectin-9 are associated with patient survival in surgically treated Hepatocellular Carcinoma independent of their intra-tumoral expression levels. Sci. Rep..

[62] Ali A., DiPersio J.F. (2024). ReCARving the future: bridging CAR T-cell therapy gaps with synthetic biology, engineering, and economic insights. Front. Immunol..

[63] Kloss C.C., Lee J., Zhang A., Chen F., Melenhorst J.J., Lacey S.F., Maus M.V., Fraietta J.A., Zhao Y., June C.H. (2018). Dominant-Negative TGF-beta Receptor Enhances PSMA-Targeted Human CAR T Cell Proliferation And Augments Prostate Cancer Eradication. Mol. Ther..

[64] Maude S.L., Laetsch T.W., Buechner J., Rives S., Boyer M., Bittencourt H., Bader P., Verneris M.R., Stefanski H.E., Myers G.D. (2018). Tisagenlecleucel in Children and Young Adults with B-Cell Lymphoblastic Leukemia. N. Engl. J. Med..

[65] Wang M., Munoz J., Goy A., Locke F.L., Jacobson C.A., Hill B.T., Timmerman J.M., Holmes H., Jaglowski S., Flinn I.W. (2020). KTE-X19 CAR T-Cell Therapy in Relapsed or Refractory Mantle-Cell Lymphoma. N. Engl. J. Med..

[66] Locke F.L., Ghobadi A., Jacobson C.A., Miklos D.B., Lekakis L.J., Oluwole O.O., Lin Y., Braunschweig I., Hill B.T., Timmerman J.M. (2019). Long-term safety and activity of axicabtagene ciloleucel in refractory large B-cell lymphoma (ZUMA-1): a single-arm, multicentre, phase 1-2 trial. Lancet Oncol..

[67] Berdeja J.G., Madduri D., Usmani S.Z., Jakubowiak A., Agha M., Cohen A.D., Stewart A.K., Hari P., Htut M., Lesokhin A. (2021). Ciltacabtagene autoleucel, a B-cell maturation antigen-directed chimeric antigen receptor T-cell therapy in patients with relapsed or refractory multiple myeloma (CARTITUDE-1): a phase 1b/2 open-label study. Lancet.

[68] Lee D.W., Santomasso B.D., Locke F.L., Ghobadi A., Turtle C.J., Brudno J.N., Maus M.V., Park J.H., Mead E., Pavletic S. (2019). ASTCT Consensus Grading for Cytokine Release Syndrome and Neurologic Toxicity Associated with Immune Effector Cells. Biol. Blood Marrow Transplant..

[69] Ghilardi G., Chong E.A., Svoboda J., Wohlfarth P., Nasta S.D., Williamson S., Landsburg J.D., Gerson J.N., Barta S.K., Pajarillo R. (2022). Bendamustine is safe and effective for lymphodepletion before tisagenlecleucel in patients with refractory or relapsed large B-cell lymphomas. Ann. Oncol..

[70] Shah B.D., Ghobadi A., Oluwole O.O., Logan A.C., Boissel N., Cassaday R.D., Leguay T., Bishop M.R., Topp M.S., Tzachanis D. (2021). KTE-X19 for relapsed or refractory adult B-cell acute lymphoblastic leukaemia: phase 2 results of the single-arm, open-label, multicentre ZUMA-3 study. Lancet.

[71] Cook M.S., King E., Flaherty K.R., Siddika K., Papa S., Benjamin R., Schurich A. (2025). CAR-T cells containing CD28 versus 4-1BB co-stimulatory domains show distinct metabolic profiles in patients. Cell Rep..

[72] Marchetti M., Corradini P., Arcaini L., Bramanti S., Di Rocco A., Ladetto M., Luminari S., Rigacci L., Zinzani P.L. (2025). A SWOT-Consensus for CAR-T in Follicular Lymphoma: Fine Tuning of Patient Journey and Selection. Hematol. Oncol..

[73] Jacobson C.A., Munoz J., Sun F., Kanters S., Limbrick-Oldfield E.H., Spooner C., Mignone K., Ayuk F., Sanderson R., Whitmore J. (2024). Real-World Outcomes with Chimeric Antigen Receptor T Cell Therapies in Large B Cell Lymphoma: A Systematic Review and Meta-Analysis. Transplant. Cell. Ther..

[74] Ayala Ceja M., Khericha M., Harris C.M., Puig-Saus C., Chen Y.Y. (2024). CAR-T cell manufacturing: Major process parameters and next-generation strategies. J. Exp. Med..

[75] Porter D.L., Hwang W.T., Frey N.V., Lacey S.F., Shaw P.A., Loren A.W., Bagg A., Marcucci K.T., Shen A., Gonzalez V. (2015). Chimeric antigen receptor T cells persist and induce sustained remissions in relapsed refractory chronic lymphocytic leukemia. Sci. Transl. Med..

[76] Turtle C.J., Hanafi L.A., Berger C., Gooley T.A., Cherian S., Hudecek M., Sommermeyer D., Melville K., Pender B., Budiarto T.M. (2016). CD19 CAR-T cells of defined CD4+:CD8+ composition in adult B cell ALL patients. J. Clin. Investig..

[77] Kater A.P., Themeli M. (2025). A burden too high for CAR T cells in CLL. Blood.

[78] Siddiqi T., Soumerai J.D., Dorritie K.A., Stephens D.M., Riedell P.A., Arnason J., Kipps T.J., Gillenwater H.H., Gong L., Yang L. (2022). Phase 1 TRANSCEND CLL 004 study of lisocabtagene maraleucel in patients with relapsed/refractory CLL or SLL. Blood.

[79] Neelapu S.S., Tummala S., Kebriaei P., Wierda W., Gutierrez C., Locke F.L., Komanduri K.V., Lin Y., Jain N., Daver N. (2018). Chimeric antigen receptor T-cell therapy - assessment and management of toxicities. Nat. Rev. Clin. Oncol..

[80] Yáñez L., Sánchez-Escamilla M., Perales M.A. (2019). CAR T Cell Toxicity: Current Management and Future Directions. HemaSphere.

[81] Gust J., Ponce R., Liles W.C., Garden G.A., Turtle C.J. (2020). Cytokines in CAR T Cell-Associated Neurotoxicity. Front. Immunol..

[82] Freyer C.W., Porter D.L. (2020). Cytokine release syndrome and neurotoxicity following CAR T-cell therapy for hematologic malignancies. J. Allergy Clin. Immunol..

[83] Kotch C., Barrett D., Teachey D.T. (2019). Tocilizumab for the treatment of chimeric antigen receptor T cell-induced cytokine release syndrome. Expert Rev. Clin. Immunol..

[84] Rejeski K., Jain M.D., Shah N.N., Perales M.A., Subklewe M. (2024). Immune effector cell-associated haematotoxicity after CAR T-cell therapy: from mechanism to management. Lancet Haematol..

[85] Yang Y., Peng H., Wang J., Li F. (2024). New insights into CAR T-cell hematological toxicities: manifestations, mechanisms, and effective management strategies. Exp. Hematol. Oncol..

[86] Alvi R.M., Frigault M.J., Fradley M.G., Jain M.D., Mahmood S.S., Awadalla M., Lee D.H., Zlotoff D.A., Zhang L., Drobni Z.D. (2019). Cardiovascular Events Among Adults Treated With Chimeric Antigen Receptor T-Cells (CAR-T). J. Am. Coll. Cardiol..

[87] Lichtenstein D.A., Schischlik F., Shao L., Steinberg S.M., Yates B., Wang H.W., Wang Y., Inglefield J., Dulau-Florea A., Ceppi F. (2021). Characterization of HLH-like manifestations as a CRS variant in patients receiving CD22 CAR T cells. Blood.

[88] Sun F., Cheng Y., Wanchai V., Guo W., Mery D., Xu H., Gai D., Siegel E., Bailey C., Ashby C. (2024). Bispecific BCMA/CD24 CAR-T cells control multiple myeloma growth. Nat. Commun..

[89] Bangolo A., Amoozgar B., Mansour C., Zhang L., Gill S., Ip A., Cho C. (2025). Comprehensive Review of Early and Late Toxicities in CAR T-Cell Therapy and Bispecific Antibody Treatments for Hematologic Malignancies. Cancers (Basel).

[90] Rejeski K., Subklewe M., Locke F.L. (2023). Recognizing, defining, and managing CAR-T hematologic toxicities. Hematology. Am. Soc. Hematol. Educ. Program.

[91] Gavriilaki E., Tzannou I., Vardi A., Tsonis I., Liga M., Gkirkas K., Ximeri M., Bousiou Z., Bouzani M., Sagiadinou E. (2025). Management strategies for CAR-T cell therapy-related toxicities: results from a survey in Greece. Front. Med..

[92] Puzanov I., Diab A., Abdallah K., Bingham C.O., Brogdon C., Dadu R., Hamad L., Kim S., Lacouture M.E., LeBoeuf N.R. (2017). Managing toxicities associated with immune checkpoint inhibitors: consensus recommendations from the Society for Immunotherapy of Cancer (SITC) Toxicity Management Working Group. J. Immunother. Cancer.

[93] Santomasso B.D., Park J.H., Salloum D., Riviere I., Flynn J., Mead E., Halton E., Wang X., Senechal B., Purdon T. (2018). Clinical and Biological Correlates of Neurotoxicity Associated with CAR T-cell Therapy in Patients with B-cell Acute Lymphoblastic Leukemia. Cancer Discov..

[94] Jagannath S., Martin T.G., Lin Y., Cohen A.D., Raje N., Htut M., Deol A., Agha M., Berdeja J.G., Lesokhin A.M. (2025). Long-Term (>/=5-Year) Remission and Survival After Treatment With Ciltacabtagene Autoleucel in CARTITUDE-1 Patients With Relapsed/Refractory Multiple Myeloma. J. Clin. Oncol..

[95] Jiang N., Yang Z., Miao H., Xing S., Wang S., Li N. (2025). Recent advances in universal chimeric antigen receptor T cell therapy. J. Hematol. Oncol..

[96] Su J., Zeng Y., Song Z., Liu Y., Ou K., Wu Y., Huang M., Li Y., Tu S. (2025). Genome-edited allogeneic CAR-T cells: the next generation of cancer immunotherapies. J. Hematol. Oncol..

[97] Cheema A.Y., Ali H.M., Maryam B., Aslam M.F., Thiagarajan P.S., Shahid D., Munir M., Najam A., Raza S., Anwer F. (2026). Under the Hood: Evidence-Based Review of Allogeneic Chimeric Antigen Receptor T Cells for Hematologic Malignancies. Transplant. Cell. Ther..

[98] Shokati A., Sanjari-Pour M., Akhavan Rahnama M., Hoseinzadeh S., Vaezi M., Ahmadvand M. (2025). Allogeneic CART progress: platforms, current progress and limitations. Front. Immunol..

[99] Fraietta J.A., Beckwith K.A., Patel P.R., Ruella M., Zheng Z., Barrett D.M., Lacey S.F., Melenhorst J.J., McGettigan S.E., Cook D.R. (2016). Ibrutinib enhances chimeric antigen receptor T-cell engraftment and efficacy in leukemia. Blood.

[100] John S., Curran K.J., Hall E.M., Keating A.K., Baumeister S.H.C., Nikiforow S., Driscoll T., Moskop A., McNerney K.O., Phillips C.L. (2025). Real-world data for tisagenlecleucel in patients with R/R B-ALL: subgroup analyses from the CIBMTR registry. Blood Adv..

[101] McNerney K.O., Schultz L.M. (2025). Tisagenlecleucel in Practice: Real-World Lessons in Pediatric and Young Adult B-ALL. Transplant. Cell. Ther..

[102] Dias J., Garcia J., Agliardi G., Roddie C. (2024). CAR-T cell manufacturing landscape-Lessons from the past decade and considerations for early clinical development. Mol. Ther. Methods Clin. Dev..

[103] Alabanza L.M., Xiong Y., Vu B., Webster B., Wu D., Hu P., Zhu Z., Dropulic B., Dash P., Schneider D. (2022). Armored BCMA CAR T Cells Eliminate Multiple Myeloma and Are Resistant to the Suppressive Effects of TGF-beta. Front. Immunol..

[104] Sheykhhasan M., Ahmadieh-Yazdi A., Vicidomini R., Poondla N., Tanzadehpanah H., Dirbaziyan A., Mahaki H., Manoochehri H., Kalhor N., Dama P. (2024). CAR T therapies in multiple myeloma: unleashing the future. Cancer Gene Ther..

[105] Ghorashian S., Kramer A.M., Onuoha S., Wright G., Bartram J., Richardson R., Albon S.J., Casanovas-Company J., Castro F., Popova B. (2019). Enhanced CAR T cell expansion and prolonged persistence in pediatric patients with ALL treated with a low-affinity CD19 CAR. Nat. Med..

[106] Thomas S., Xue S.A., Bangham C.R.M., Jakobsen B.K., Morris E.C., Stauss H.J. (2011). Human T cells expressing affinity-matured TCR display accelerated responses but fail to recognize low density of MHC-peptide antigen. Blood.

[107] Shahid S., Prockop S.E., Flynn G.C., Mauguen A., White C.O., Bieler J., McAvoy D., Hosszu K., Cancio M.I., Jakubowski A.A. (2025). Allogeneic off-the-shelf CAR T-cell therapy for relapsed or refractory B-cell malignancies. Blood Adv..

[108] Qiu S., Chen J., Wu T., Li L., Wang G., Wu H., Song X., Liu X., Wang H. (2024). CAR-Toner: an AI-driven approach for CAR tonic signaling prediction and optimization. Cell Res..

[109] Bäckel N., Hort S., Kis T., Nettleton D.F., Egan J.R., Jacobs J.J.L., Grunert D., Schmitt R.H. (2023). Elaborating the potential of Artificial Intelligence in automated CAR-T cell manufacturing. Front. Mol. Med..

[110] Gardner R.A., Finney O., Annesley C., Brakke H., Summers C., Leger K., Bleakley M., Brown C., Mgebroff S., Kelly-Spratt K.S. (2017). Intent-to-treat leukemia remission by CD19 CAR T cells of defined formulation and dose in children and young adults. Blood.

[111] Wang Z., Lu Y., Liu Y., Mou J., Liu X., Chen M., Wang Y., Xu Y., Rao Q., Xing H. (2023). Novel CD123xCD33 bicistronic chimeric antigen receptor (CAR)-T therapy has potential to reduce escape from single-target CAR-T with no more hematotoxicity. Cancer Commun..

[112] Luanpitpong S., Klaihmon P., Janan M., Kungwankiattichai S., Owattanapanich W., Kunacheewa C., Chanthateyanonth S., Donsakul N., U-Pratya Y., Warindpong T. (2024). Point-of-care manufacturing of anti-CD19 CAR-T cells using a closed production platform: Experiences of an academic in Thailand. Mol. Ther. Oncol..

[113] Tix T., Alhomoud M., Shouval R., Cliff E.R.S., Perales M.A., Cordas Dos Santos D.M., Rejeski K. (2024). Second Primary Malignancies after CAR T-Cell Therapy: A Systematic Review and Meta-analysis of 5,517 Lymphoma and Myeloma Patients. Clin. Cancer Res..

[114] Abou-El-Enein M. (2024). The Fate(s) of CAR T-Cell Therapy: Navigating the Risks of CAR+ T-Cell Malignancy. Blood Cancer Discov..

[115] Levine B.L., Pasquini M.C., Connolly J.E., Porter D.L., Gustafson M.P., Boelens J.J., Horwitz E.M., Grupp S.A., Maus M.V., Locke F.L. (2024). Unanswered questions following reports of secondary malignancies after CAR-T cell therapy. Nat. Med..

[116] Olaghere J., Williams D.A., Farrar J., Büning H., Calhoun C., Ho T., Inamdar M.S., Liu D., Makani J., Nyarko K. (2025). Scientific Advancements in Gene Therapies: Opportunities for Global Regulatory Convergence. Biomedicines.

